# Insights from an unfortunate FDG contamination

**DOI:** 10.1007/s00259-026-08018-7

**Published:** 2026-07-01

**Authors:** Wouter V. Vogel, Sylke Woudsma, Rick Schilder, Helin Yigit, Miranda Kossen

**Affiliations:** 1 Department of Nuclear Medicine, NKI-AVL, Amsterdam, Netherlands; 2 Department of Radiation safety, NKI-AVL, Amsterdam, Netherlands; 3 Department of Radiation Oncology, The Netherlands Cancer Institute – Antoni van Leeuwenhoek (NKI-AVL), Plesmanlaan 121, Amsterdam, 1066 CX The Netherlands

**Figure:**
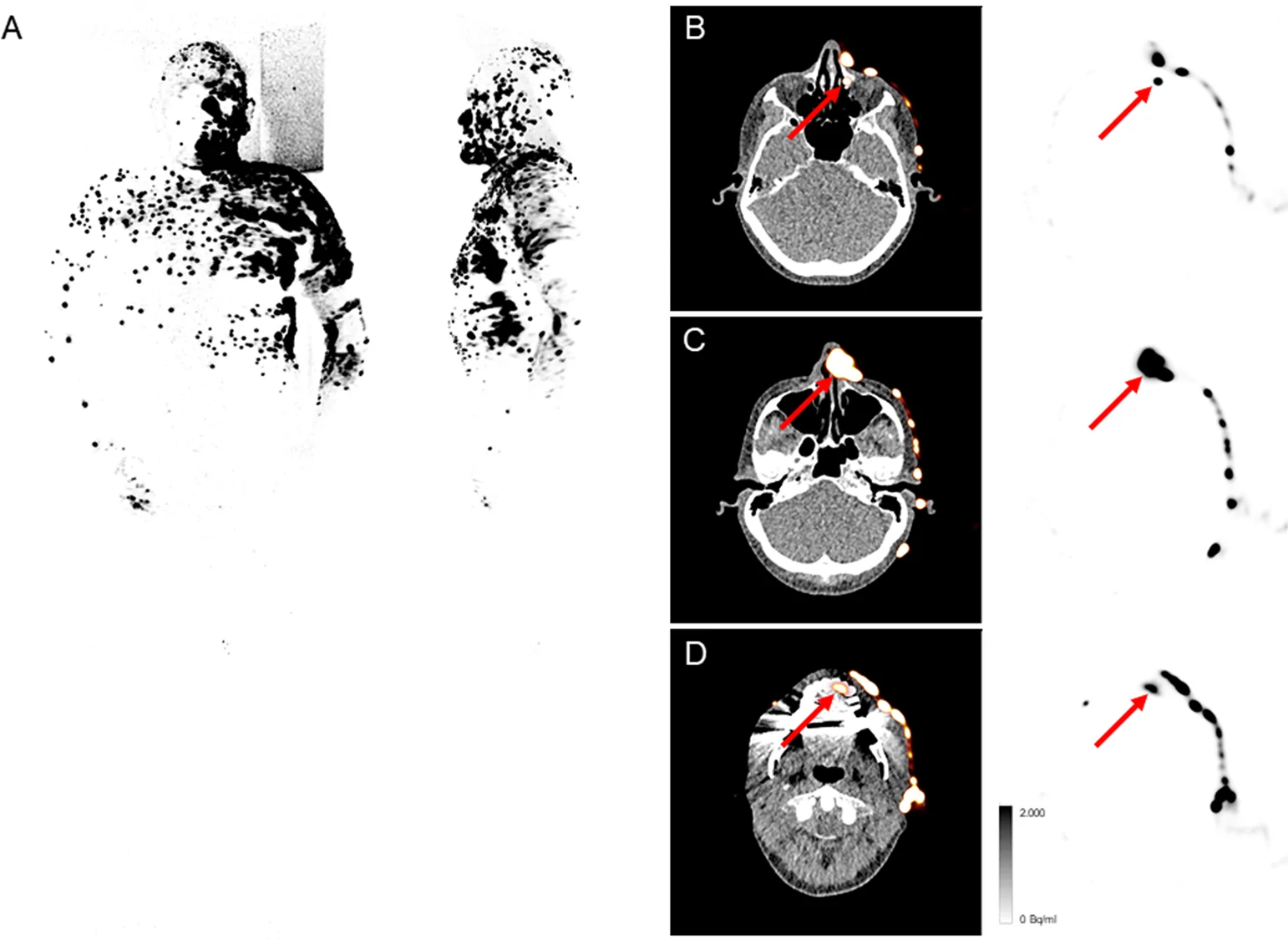

An uncapped lead-shielded syringe containing 106 MBq [^18^F]-FDG in 0.8 ml was dropped on the technician’s lap just prior to injection. It landed on the plunger, spraying droplets in the air. Both the technician and patient noticed some droplets landing on their face and clothes. Radiation measurements were positive including their surroundings, making it hard to localize and clean individual droplets. The technician showered and changed clothes, and was confirmed clean afterwards. The room was sealed for cleaning after decay.

The main challenge was decontamination of the patient, a 70-year-old male with a history of recurrent melanoma. He first washed his hands and face with water and decontamination soap (S4FE-D decon spray), but radiation measurements remained positive. Because of uncertainty about the extent, 30 min after the event a PET-scan with ultra-lowdose CT was acquired (Siemens Vision Quadra) to guide further cleaning advice. This revealed remaining contamination of face, arms and clothes, more extensive than previously thought (A, MIP from anterior and lateral). In addition, the images depicted some FDG internalizing through the eyes (B, nasolacrimal duct), nose (C, nasal mucosa) and mouth (D, tip of tongue). Based on these findings, the standard instructions to change clothes after showering were expanded with rinsing of mouth, nose and eyes. He was rescheduled for a new FDG injection and scan the next day.

This unfortunate event can provide some insights about best practice for patient decontamination. The extent of droplet contamination is easily underestimated. Local skin cleaning by an untrained patient may not always be effective, showering and changing clothes is generally indicated. Tracer in cutaneous drops may rapidly internalize through multiple routes, especially in the facial area. When in doubt about the extent of contamination, imaging may help to guide personalized cleaning advice.

## Data Availability

The data presented in the current study are available from the corresponding author on reasonable request.

